# Nutcracker syndrome, conservative approach: a case report

**DOI:** 10.1093/jscr/rjac423

**Published:** 2022-10-23

**Authors:** Muheilan Muheilan, Anna Walsh, Frank O’Brien, David Tuite

**Affiliations:** Department of Urology, Cork University Hospital, Cork, Ireland; Department of Urology, Cork University Hospital, Cork, Ireland; Department of Urology, Cork University Hospital, Cork, Ireland; Department of Radiology, Cork University Hospital, Cork, Ireland

## Abstract

The nutcracker phenomenon (NCP) refers to the compression of the left renal vein, most commonly between the aorta and the superior mesenteric artery (SMA). Nutcracker syndrome (NCS) should be limited to patients who present with the characteristic clinical signs and symptoms alongside diagnostic imaging of the anatomy associated with the syndrome. We report a case of NCS presenting with painless visible hematuria and left flank pain. Imaging showed a left renal vein stenosis at the origin of the SMA with collateralization. Diagnosis of NCP is made by a variety of imaging techniques; approaches to the treatment of NCS include conservative methods, open surgical, laparoscopic or endovascular techniques. Correlation with symptoms, laboratory results and excluding other causes continues to be important in the workup of NCS. Collaboration with the establishment of an International Consortium database will aid in the understanding of this rare disease.

## INTRODUCTION

The nutcracker phenomenon (NCP) refers to the compression of the left renal vein (LRV), most commonly between the aorta and the superior mesenteric artery (SMA) [1].

The term nutcracker syndrome (NCS) is used for patients with clinical symptoms and signs, whereas the NCP describes the anatomical variation without symptoms [2, 3].

The prevalence of this condition has been reported as higher in females; however, further studies have shown that it is equally prevalent among both genders. The venous reflux is responsible for the typical clinical scenario comprising left flank pain and chronic abdominal pain. In women, it may cause pelvic congestion syndrome. In men, the syndrome can manifest in a similar manner and has been described as one of the causes of varicocele [4–8].

We report a conservatively managed case of NCS presented with painless visible hematuria, clot retention and left flank pain.

## CASE PRESENTATION

A 42-year-old female patient was admitted via the emergency department with macroscopic painless hematuria and clot retention accompanied by left flank pain without previous lower urinary tract symptoms.

**Figure 1:**
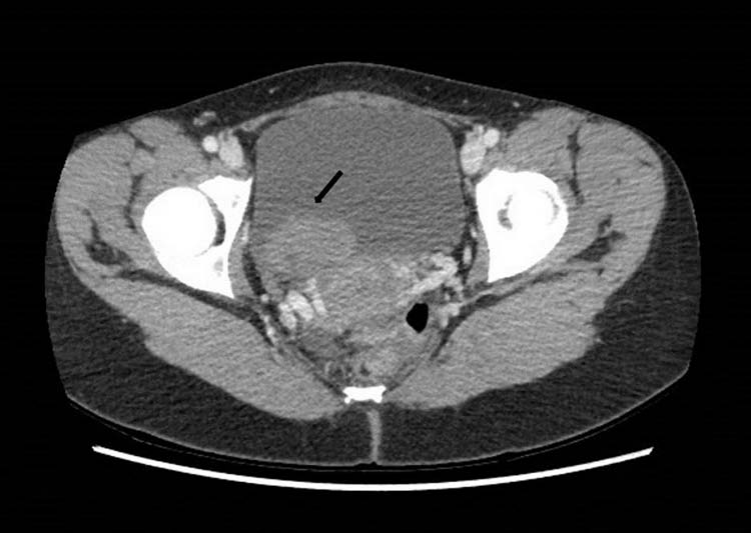
Computed tomography on presentation showing a bladder mass (arrow).

**Figure 2:**
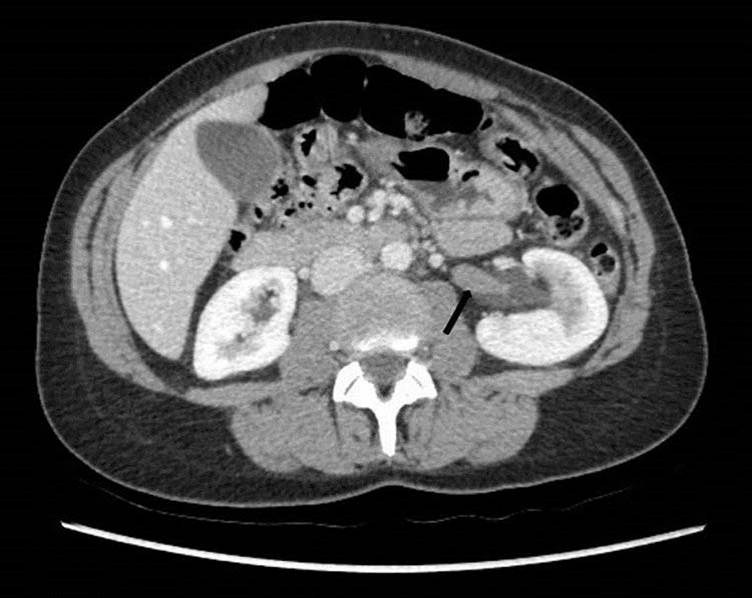
Computed tomography on presentation showing the hypodense filling defect extending from the left renal pelvis to the proximal ureter with associated hydronephrosis (arrow).

The patient received a bladder washout and continued on continuous bladder irrigation. She was afebrile and normotensive. Laboratory findings showed raised creatinine at 140 mmol/L, Hb was 13 gm/dL with subsequent drop to 9 gm/dL during the admission. Urine culture didn’t show signs of infection. Computed Tomography (CT) abdomen and pelvis with contrast demonstrated a solid mass in the bladder and a hypodense filling defect extending from the left renal pelvis to the proximal ureter with hydronephrosis (Figs 1 and 2).

We performed a rigid cystoscopy and left ureteroscopy 2 days later, the bladder contained a large clot without tumor and the left ureteric orifice was dilated with a clot seen coming out of it. The left ureteroscopy was limited because of hematuria but no mass seen and JJ stent inserted.

**Figure 3:**
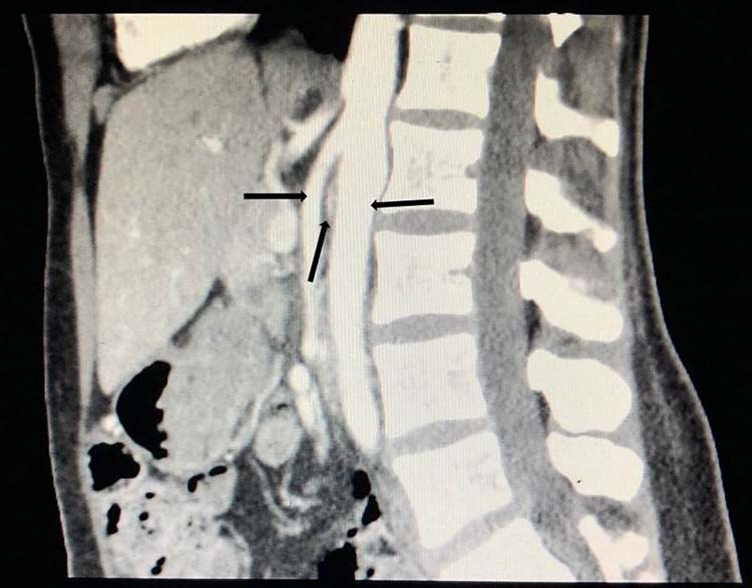
Computed tomography angiogram on presentation, sagittal view showing origin of SMA (left arrow) and the aorta (right arrow) with the compressed LRV in the narrow angle (middle arrow).

**Figure 4:**
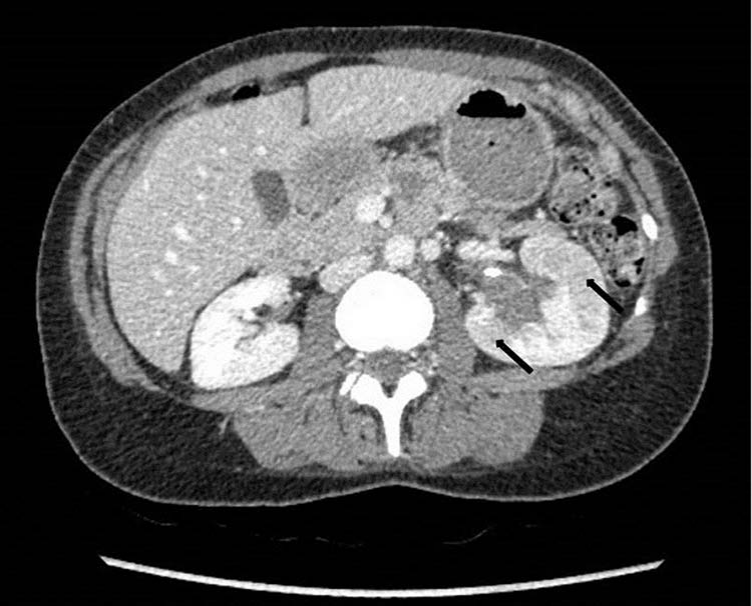
Computed tomography on presentation showing multifocal areas of hypoattenuation throughout the left renal cortex (arrows).

**Figure 5:**
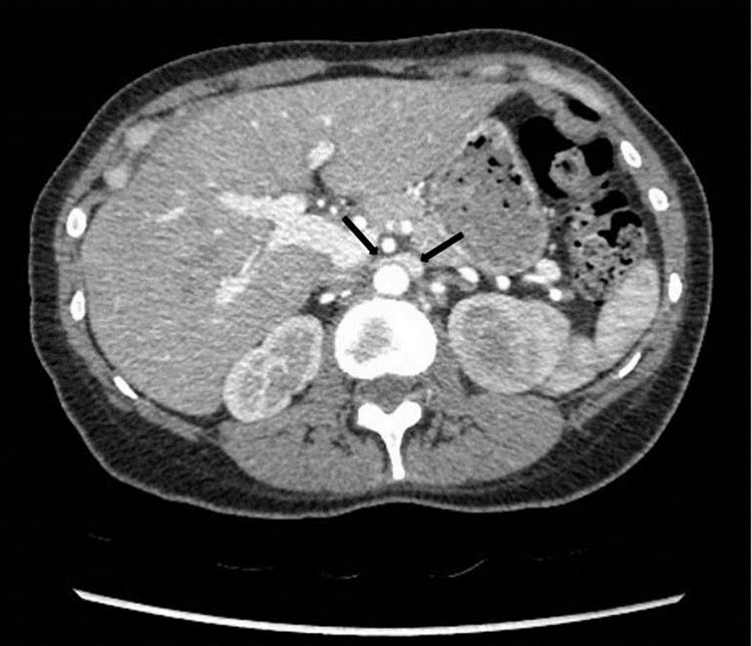
Computed tomography angiogram on presentation showing pre-compressed LRV (right arrow); and compressed LRV (left arrow).

To investigate the bleeding source, she subsequently had a CT renal angiogram 3 days later that showed a narrow angle between origin of SMA and the aorta (Fig. 3) with multifocal areas of hypoattenuation throughout the left renal cortex (Fig. 4). It showed a compression ratio of 3.25 (diameter of pre-compressed vein =6.5 mm; diameter of compressed vein =2 mm) (Fig. 5).

**Figure 6:**
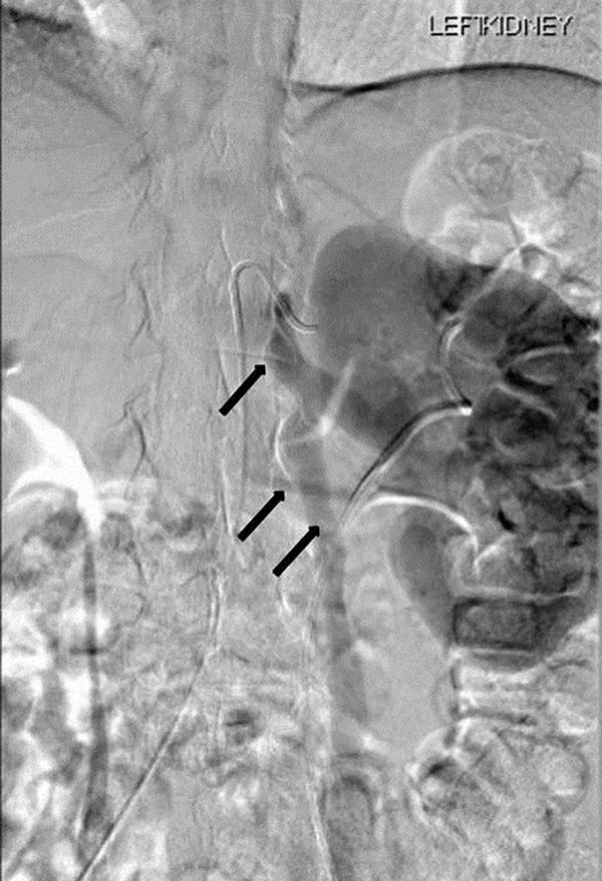
Renal angiography showing thin trickle of contrast passing through the LRV into the inferior vena cava (IVC) (top arrow), paraspinal collateral (middle arrow) and retrograde reflux of contrast into left ovarian vein (bottom arrow).

Renal angiography performed 1 day after the CT angiogram demonstrated no arteriovenous malformation. It showed a thin trickle of contrast passing through the LRV into the IVC (Fig. 6).

**Figure 7:**
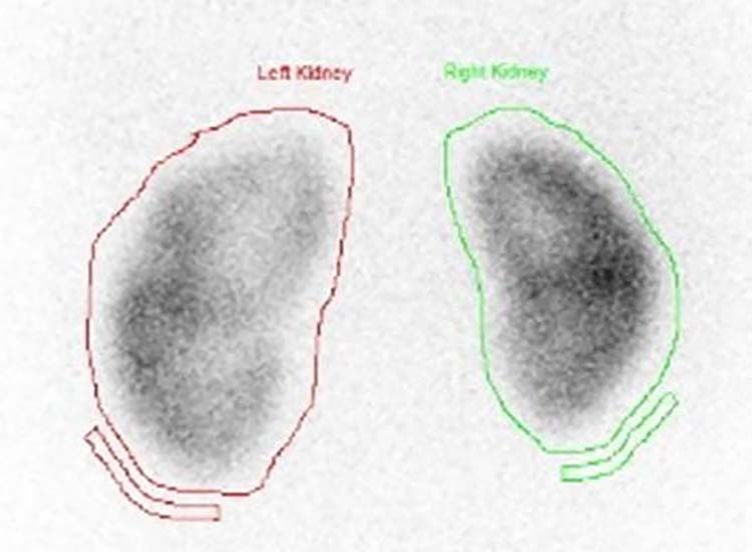
DMSA scan on presentation showing symmetrical bilateral uptake of the radioisotope with slight enlargement of the left kidney with patchy uptake in the renal cortex.

A dimercaptosuccinic acid (DMSA) scan performed 4 days later showed patchy uptake in the renal cortex. However, there was symmetrical bilateral uptake of the radioisotope (Fig. 7). Urine protein creatinine ratio was insignificantly elevated (63).

Patient discharged after 10 days of hospital stay, with complete resolution of symptoms, but was readmitted 10 days later with macroscopic hematuria and remained on bladder irrigation for several days and settled again with conservative management after a week.

**Figure 8:**
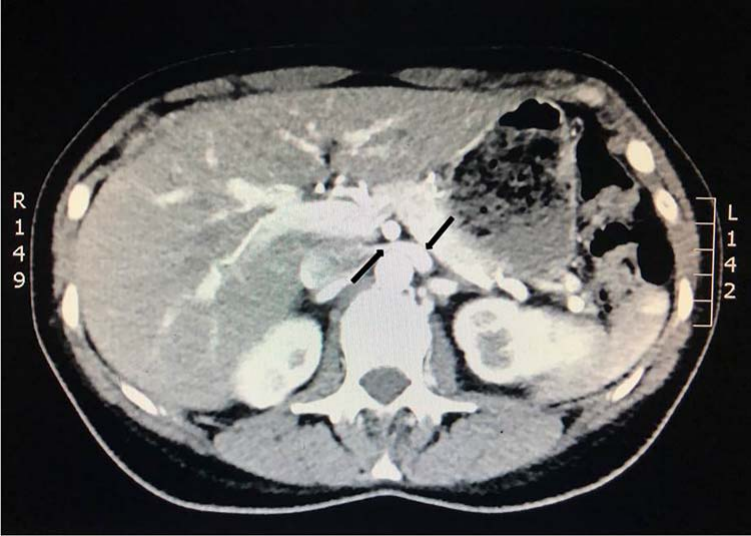
Computed tomography angiogram 6 months later showing pre-compressed LRV (right arrow); and compressed LRV (left arrow), but resolution of the congestion and swelling of the left kidney.

She underwent a flexible ureterorenoscopy 2 months later which further demonstrated no cause for hematuria within the collecting system. An ECHO cardiography was performed to out rule an embolic event to the kidney due to the patchy perfusion noted and was normal. A repeat CT angiogram showed resolution of the congestion of the left kidney, but a narrow LRV as before (Fig. 8).

### Follow-up

On follow-up over 44 months, she has remained symptom free with no further hematuria or flank pain, normal kidney function test and no drop of haemoglobin.

## DISCUSSION

NCS can be classified clinically into two types: typical, with urological manifestations including gross hematuria and orthostatic proteinuria, with or without flank pain, and atypical, with non-urologic manifestations including fatigue, orthostatic intolerance, dysmenorrhea and dyspareunia in women, and varicocele in men [7].

Diagnosis of nutcracker anatomy can be made by a variety of imaging techniques, including magnetic resonance angiography, CT angiography, conventional Doppler ultrasonography, phlebography with venous pressure measurement and intravascular ultrasound.

Doppler ultrasound has a high sensitivity and specificity of 69–90% and 89–100%, respectively. Although it is recommended as the first diagnostic investigation, its index and range of values in NCS diagnosis are highly variable. This is because findings vary depending on patient position, as well as technical difficulties resulting from the very small sampling rea [2].

A strong correlation between the degree of LRV compression on CT in diagnosing NCS was identified. A pre-compression to compression ratio of the LRV over 2.25 is reported to have a greater than 91% specificity and sensitivity for NCS [8].

The management of NCS is determined by symptom severity; treatment of NCS has traditionally been through conservative methods, surgery or endovascular stents. Conservative treatment is suggested for patients with tolerable symptoms. Surgical options include but are not limited to LRV transposition, LRV transposition with patch venoplasty, patch venoplasty without LRV transposition, LRV transposition with saphenous vein cuff, gonadal vein transposition and saphenous vein bypass. Among these, LRV transposition is the most common and effective approach [9].

One institutional review of 16 patients treated conservatively; clinical improvement occurred in 11 with total relief of symptoms in 2 patients and partial relief in 9 patients. There was no relief of symptoms in five patients following a mean follow-up of 41.2 months [10].

In a systematic review on management of NCS, 17 references were reviewed. Eight (47%) described the open surgical approach. The LRV transposition was the most commonly reported technique, followed by renal autotransplantation. Seven (41.11%) described the endovascular technique of stent implantation, and two (11.7%) described the minimally invasive laparoscopic extravascular stent implantation [11]. The longevity and durability of these techniques have not been shown and are compounded further by the general young age of the patients.

## CONCLUSIONS

NCS is a rare entity. The management of NCS depends upon the clinical presentation and the severity of the symptoms. Multiple techniques have been developed for the treatment of this condition.

Our patient is being managed conservatively for hematuria and loin pain with relief from symptoms.

Correlation with symptoms, laboratory results and excluding other causes continues to be important in the workup of NCS. Collaboration with the establishment of an International Consortium database will aid in the understanding of this rare disease.

## CONSENT FOR PUBLICATION

Written informed consent was obtained from the patient for publication of this case report and accompanying images.

## GUARANTORS

Muheilan Muheilan and Frank O’Brien.

## CONFLICT OF INTEREST STATEMENT

The authors declare that they have no conflict of interest.

## FUNDING

None.

## AUTHORS’ CONTRIBUTION

All authors have contributed significantly and as following:

Muheilan Muheilan: Literature review, case presentation, submitting, corresponding and main author.

Anna Walsh: Patient follow-up.

Frank O’Brien: Principal urologist treating the patient and review of final manuscript.

David Tuite: Radiology interpretation, literature review and review of final manuscript.
